# Inguinal hernia repair: The total picture

**DOI:** 10.4103/0972-9941.27727

**Published:** 2006-09

**Authors:** Tehemton E Udwadia

**Affiliations:** Department of Minimal Access Surgery, P. D. Hinduja National Hospital, Veer Savarkar Marg, Mahim, Mumbai - 400 016, India

“Groin hernia repair does not have the glamour of a Whipple or of a heart transplant, but in terms of preserving years of useful life, in sheer volume, is one of the most important surgical procedures.”

Dr. Jonathan E. Rhoades

Repair of inguinal hernia is one of the commonest surgical procedures worldwide, irrespective of country, race or socio-economic status and constitutes a major health-care drain in every country.

There are three landmarks in the history of repair of inguinal hernia.

**Table:** 

1. Tissue repair	Eduardo Bassini	1888
2. Onlay mesh (tension-free) repair	Irving Lichtenstein	1984
3. Laparoscopic hernia repair	Ger, Shultz Corbitt etc	1990

Bassini in 1988 posted a milestone in the history of not only hernia surgery but of all surgery when he reported a reduction in the recurrence rate from 100 to 10% with his operation which was a unique combination of understanding of anatomy and application of surgical thinking and technique.[1] This 10% recurrence rate was achieved at a period without antibiotics, primitive anaesthesia and when patients suffered their hernia to giant size before submitting to surgery. Over nine decades Bassini's tissue repair procedures – with several modifications (Halsted, McVay, Tanner, Shouldice….) has helped preserve useful life in hundreds of thousands cases. While most “herniologists” view tissue repair as a method in disrepute to be discarded, its very economical cost structure makes it even today the commonest form of hernia repair in most part of the developing world and even in Canada tissue repair (Shouldice) accounts for 25% of all inguinal hernia repair.[2]

The problems associated with tension in tissue repair specially with wide defects - pain, prolonged recovery time, recurrence - prompted surgeons to seek some form of tension-free repair. In my 50 years in surgery I have witnessed and practiced several such procedures - Mair (Skin), floss silk, steel and tantalum mesh - all to be relegated to surgical history when tension-free mesh repair came on the scene.

It is worth recording that the first mesh plasty in clinical work was reported by Usher in 1958 when he used mesh with elasticized nylon on humans with no prior experimental work.[3] The tension-free onlay mesh repair is invariably linked to Lichtenstein whose work and progress over two decades culminated in the final Lichtenstein repair.[4,5]

Aware that Bassini's original article was open to misinterpretation and modification, Lichtenstein crystallized his procedure to a few simple essentials - local anaesthesia, adequate mesh size, inferomedial corner to overlap the pubic tubercle, overlap of the mesh lateral to the cord, loose sutures between mesh and tissue and early if not immediate mobilization. Tension-free mesh hernia repair by virtue of low degree and duration of pain, early return to normal activity, low recurrence rate is the accepted method of choice even in the developing world, whenever economics permit the cost of the mesh.

The advent of laparoscopic cholecystectomy opened the floodgates for laparoscopy in all surgery. Even before the era of laparoscopic surgery Ger had advocated laparoscopic repair of hernia.[6] Given the added benefit of reduced pain and early mobilization it is a good procedure in expert hands. However, these benefits are outweighed by several factors like its longer learning curve, higher cost, need for general anaesthesia. Today laparoscopic repair accounts for 5 to 15% of all hernia repair in the developed world and a miniscule percentage in the developing world. Any commonly performed procedure should be evaluated on several essential parameters. Table 1 shows the comparison of the parameters of these three methods of repair

**Table 1:** Comparison of tissue, open tension-free mesh and laparoscopic repair for inguinal hernia

	Tissue repair	Open mesh	Laparoscopic
Applicability in every case	X	√	X
Clear cut anatomy	√√	√√	√
Simple / safe anaesthesia (local)	√	√√	X
Ease of operative procedure	√	√√	√
Easily leant by the junior staff	√	√√	X
Short learning curve	√	√√	X
Short duration of procedure	√	√√	√
Simple routine equipment	√	√√	X
Short or no time in recovery	√	√√	√√
No major life-threatening complications	√√	√√	√
Minimal discomfort / pain	√	√√	√
Early return to activity	X	√	√√
Low cost	√	√√	√
Low recurrence rate	√	√√	√√
Overall score	17	26	14

Papers on hernia repair at conferences and publication on hernia repair in journals are made by herniologists who work in ideal conditions in developed countries and urban centres in the developing world. Cocooned in their sophistication they kneel at the alter of what they believe is “evidence based medicine”. Sadly, most do not know (and some do not care) about the problems of hernia repair in 70% of the world population, where for example, in East Africa patients with strangulated hernia get no treatment.[7]

In 1998, I was interested and excited to learn that surgeons in rural areas of India were doing tension-free repairs using indigenous mesh which was autoclavable (the only mean of sterilization to them) and had similar weave, tensile strength, chemical composition and biological response as commercially available mesh.[8] God in his wisdom made mosquitoes endemic in the developing world, necessitating the manufacturing of cheap mosquito-net for mass use.

As the editor of Indian Journal of Surgery I accepted for publication the article on “Preliminary multicentric trial of cheap indigenous mosquito net cloth for tension free hernia repair”. By doing so I invited the wrath of Heads of Department in prestigious Indian teaching hospitals who questioned my Editorial propriety in accepting an article with no animal toxicology study, no controlled trial and questionable follow up. I gently explained my acceptance of the article by reminding them that the first clinical mesh study was done without experimental work in 1958, that the Heads of Department were in a stronger position to do such a study than the rural surgeon, that research not applicable to the needs of a developing country was unethical[9] and that follow-up in a village or small town was far more reliable than urban cities because small town surgeon knew each one of his patient for years and by name.

I am aware that like those Heads of Department, all herniologists who talk and write on hernia will find the use of this simple cheap mesh (cost ratio 1:2000) surgical blasphemy. History of hernia repair teaches us that Bassini, Shouldice, Lichtenstein much after they advocated their procedure were held in ridicule. In 1972, the role of laparoscopy in surgery was blasphemy and both Semm who performed the first laparoscopic appendicectomy and Muhe who performed the first laparoscopic cholecystectomy were ostracized for years for their blasphemy. Appropriately George Bernard Shaw wrote “Most truths start off as blasphemy”. Time will tell if this ingenious work of rural Indian surgeons is a truth. If it is, it will be a true landmark in the history of hernia repair, for it will provide all the benefits of the Lichtenstein procedure, at virtually no cost for the mesh, underscoring the true role of surgery – good surgical care too all people, in all places.

## CONCLUSION

All methods of hernia repair if done with gentle care (which local anaesthesia helps to cultivate), indivisualized selection and attention to detail by experts are good. Rather than take a fundamentalists stand on the superiority of one method over any other, let us imbibe the wisdom of Sir John Bruce.

“The final word on hernia will probably never be written. In collecting, assimilating and distilling the wisdom of today we must provide a base from which further advances may be made.”

Sir John Bruce
